# Supplementary material for: Modified Ant Colony Optimization as a Means for Evaluating the Variants of the City Railway Underground Section
Source: Int J Environ Res Public Health. 2023 Mar 11;20(6):4960. doi: 10.3390/ijerph20064960 (PMC10048943; doi:10.3390/ijerph20064960)
Supplement: Supplementary file 1 [file ijerph-20-04960-s001.zip › ijerph-2218801-supplementary.pdf]

Supplementary Materials

Python code. Used to determine the effort required to travel the indicated route. Below is shown the code for route number one. For the other routes, only the effort matrix E is different.

```python
import numpy as np
from numpy import inf

#effort matrix

E = np.array([[0,0.9,1.1,2.0,1.9,4.5,7.3,8.2,10.9]
          ,[0.9,0,0.7,1.9,2,6,13.4,15.5,23.5]
          ,[1.1,0.7,0,0.6,0.9,2.5,4.8,5.7,9.1]
          ,[2,1.9,0.6,0,0.7,2.6,4.9,6.2,10.2]
          ,[1.9,2,0.9,0.7,0,1.1,2.3,3.1,5.6]
          ,[4.5,6,2.5,2.6,1.1,0,2.5,4.6,9.8]
          ,[7.3,13.4,4.8,4.9,2.3,2.5,0,5.3,17.4]
          ,[8.2,15.5,5.7,6.2,3.1,4.6,5.3,0,10.8]
          ,[10.9,23.5,9.1,10.2,5.5,9.8,17.4,10.8,0]])

iteration = 10
n_ants = 50
n_citys = 9

# intialization part

m = n_ants
n = n_citys
e = 0.5          #evaporation rate
alpha = 2      #pheromone factor
beta = 1        #visibility factor

#calculating the visibility of the next station visibility(i,j)=1/d(i,j)

visibility = 1/J
visibility[visibility == inf ] = 0

#initializing pheromne present at the paths to the stations

pheromne = np.ones((m,n))

#initializing the route of the ants with size route(n_ants,n_citys)

rute = np.ones((m,n))

for ite in range(iteration):

    rute[:,0] = 1

    for i in range(m):

        temp_visibility = np.array(visibility)

        for j in range(n-1):

            combine_feature = np.zeros(5)
            cum_prob = np.zeros(5)

            cur_loc = int(rute[i,j]-1)

            temp_visibility[:,cur_loc] = 0

            p_feature = np.power(pheromne[cur_loc,:],beta)
            v_feature = np.power(temp_visibility[cur_loc,:],alpha)

            p_feature = p_feature[:,np.newaxis]
            v_feature = v_feature[:,np.newaxis]

            combine_feature = np.multiply(p_feature,v_feature)

            total = np.sum(combine_feature)

            probs = combine_feature/total

            cum_prob = np.cumsum(probs)

```

```
            r = np.random.random_sample()

            city = np.nonzero(cum_prob>r)[0][0]+1


            rute[i,j+1] = city

    rute_opt = np.array(rute)

    dist_cost = np.zeros((m,1))

    for i in range(m):

        s = 0
        for j in range(n-1):

            s = s + d[int(rute_opt[i,j])-1,int(rute_opt[i,j+1])-1]

        dist_cost[i]=s

    dist_min_loc = np.argmin(dist_cost)
    dist_min_cost = dist_cost[dist_min_loc]

    best_route = rute[dist_min_loc,:]
    pheromne = (1-e)*pheromne

    for i in range(m):
        for j in range(n-1):
            dt = 1/dist_cost[i]
            pheromne[int(rute_opt[i,j])-1,int(rute_opt[i,j+1])-1] =
            = pheromne[int(rute_opt[i,j])-1,int(rute_opt[i,j+1])-1] + dt

print('------------------------------------------------------------')
print('route of all the ants at the end :')
print(rute_opt)
print()
print('best route :',best_route)
print()
print('ant effort:', int(dist_min_cost[0]) + d[int(best_route[-2])-1,0])

------------------------------------------------------------
route of all the ants at the end :
[[1. 2. 3. 4. 5. 6. 7. 8. 9.]
 [1. 2. 3. 4. 5. 6. 7. 8. 9.]
 [1. 2. 3. 4. 5. 6. 7. 8. 9.]
 [1. 2. 3. 4. 5. 6. 7. 8. 9.]
 [1. 2. 3. 4. 5. 6. 7. 8. 9.]
 [1. 2. 3. 4. 5. 6. 7. 8. 9.]
 [1. 2. 3. 4. 5. 6. 7. 8. 9.]
 [1. 2. 3. 4. 5. 6. 7. 8. 9.]
 [1. 2. 3. 4. 5. 6. 7. 8. 9.]
 [1. 2. 3. 4. 5. 6. 7. 8. 9.]
 [1. 2. 3. 4. 5. 6. 7. 8. 9.]
 [1. 2. 3. 4. 5. 6. 7. 8. 9.]
 [1. 2. 3. 4. 5. 6. 7. 8. 9.]
 [1. 2. 3. 4. 5. 6. 7. 8. 9.]
 [1. 2. 3. 4. 5. 6. 7. 8. 9.]
 [1. 2. 3. 4. 5. 6. 7. 8. 9.]
 [1. 2. 3. 4. 5. 6. 7. 8. 9.]
 [1. 2. 3. 4. 5. 6. 7. 8. 9.]
 [1. 2. 3. 4. 5. 6. 7. 8. 9.]
 [1. 2. 3. 4. 5. 6. 7. 8. 9.]
 [1. 2. 3. 4. 5. 6. 7. 8. 9.]
 [1. 2. 3. 4. 5. 6. 7. 8. 9.]
 [1. 2. 3. 4. 5. 6. 7. 8. 9.]
 [1. 2. 3. 4. 5. 6. 7. 8. 9.]
 [1. 2. 3. 4. 5. 6. 7. 8. 9.]
 [1. 2. 3. 4. 5. 6. 7. 8. 9.]
 [1. 2. 3. 4. 5. 6. 7. 8. 9.]
 [1. 2. 3. 4. 5. 6. 7. 8. 9.]
 [1. 2. 3. 4. 5. 6. 7. 8. 9.]
 [1. 2. 3. 4. 5. 6. 7. 8. 9.]
 [1. 2. 3. 4. 5. 6. 7. 8. 9.]
 [1. 2. 3. 4. 5. 6. 7. 8. 9.]
 [1. 2. 3. 4. 5. 6. 7. 8. 9.]
 [1. 2. 3. 4. 5. 6. 7. 8. 9.]
 [1. 2. 3. 4. 5. 6. 7. 8. 9.]
```

```
 [1. 2. 3. 4. 5. 6. 7. 8. 9.]
 [1. 2. 3. 4. 5. 6. 7. 8. 9.]
 [1. 2. 3. 4. 5. 6. 7. 8. 9.]
 [1. 3. 4. 5. 6. 7. 8. 9. 2.]
 [1. 2. 3. 4. 5. 6. 7. 8. 9.]
 [1. 2. 3. 4. 5. 6. 7. 8. 9.]
 [1. 2. 3. 4. 5. 6. 7. 8. 9.]
 [1. 2. 3. 4. 5. 6. 7. 8. 9.]
 [1. 2. 3. 4. 5. 6. 8. 9. 7.]
 [1. 2. 3. 4. 5. 6. 7. 8. 9.]
 [1. 2. 3. 4. 5. 6. 7. 8. 9.]
 [1. 2. 3. 4. 5. 6. 7. 8. 9.]
 [1. 2. 3. 4. 5. 6. 7. 8. 9.]
 [1. 2. 3. 4. 5. 6. 7. 8. 9.]
 [1. 2. 3. 4. 5. 6. 7. 8. 9.]]

best route : [1. 2. 3. 4. 5. 6. 7. 8. 9.]

ant effort: 30.2
```
